# Challenges and developments in universal vaccine design against SARS-CoV-2 variants

**DOI:** 10.1038/s41541-022-00597-4

**Published:** 2022-12-19

**Authors:** Fangxin Zhao, Xiaodong Zai, Zhiling Zhang, Junjie Xu, Wei Chen

**Affiliations:** 1 Laboratory of Vaccine and Antibody Engineering, Beijing Institute of Biotechnology, Beijing, 10071 China; 2 School of Medicine, Zhejiang University, Hangzhou, 310058 China; 3 College of pharmacy, Nanjing University of Chinese Medicine, Nanjing, 210023 China

**Keywords:** Biotechnology, Vaccines, Infectious diseases

## Abstract

The emergence of severe acute respiratory syndrome coronavirus 2 (SARS-CoV-2) had become a global concern because of its unexpectedly high pathogenicity and transmissibility. SARS-CoV-2 variants that reduce the immune protection elicited from previous vaccination or natural infection raise challenges in controlling the spread of the pandemic. The development of universal vaccines against these variants seems to be a practical solution to alleviate the physical and economic effects caused by this disease, but it is hard to achieve. In this review, we describe the high mutation rate of RNA viruses and dynamic molecular structures of SARS-CoV-2 variants in several major neutralizing epitopes, trying to answer the question of why universal vaccines are difficult to design. Understanding the biological basis of immune evasion is crucial for combating these obstacles. We then summarize several advancements worthy of further study, including heterologous prime-boost regimens, construction of chimeric immunogens, design of protein nanoparticle antigens, and utilization of conserved neutralizing epitopes. The fact that some immunogens can induce cross-reactive immune responses against heterologous coronaviruses provides hints for universal vaccine development. We hope this review can provide inspiration to current universal vaccine studies.

## Introduction to SARS-CoV-2 pandemic

The severe acute respiratory syndrome coronavirus 2 (SARS-CoV-2) causing coronavirus disease 2019 (COVID-19) was first reported in Wuhan, China in December 2019^1,2^. Since the initial outbreak of COVID-19, it has been declared a pandemic by World Health Organization (WHO) and has taken the lives of over 6.5 million people globally (https://covid19.who.int/), bringing serious threats to economic and social stability.

SARS-CoV-2 belongs to the *Orthocoronavirinae* subfamily, which can be subdivided into *Alphacoronavirus, Betacoronavirus, Gammacoronavirus*, and *Deltacoronavirus* genera based on their genomic structures^3^. Coronaviruses mainly caused mild respiratory symptoms until SARS-CoV, first reported in 2003, which resulted in a 10% mortality^4,5^. Both SARS-CoV and SARS-CoV-2 are members of the subgenus *Sarbecovirus* of the genus *Betacoronavirus*. Although the protein structure of the immunodominant region of SARS-CoV-2 is similar to that of SARS-CoV, the antibodies elicited from a particular strain are more prone to have cross-reaction instead of cross-neutralization activity to a heterologous virus^6,7^. Middle East respiratory syndrome coronavirus (MERS-CoV), another highly pathogenic *Betacoronavirus* (subgenus *Merbecovirus*) that still circulates, was discovered in Saudi Arabia in 2012^8^. The emergence of these viruses and their serious consequences make coronavirus a main focus of human disease prevention research.

Consistent with many other coronaviruses, SARS-CoV-2 utilizes its surface spike (S) glycoprotein to enter into the host cell^9–11^. The homotrimer S can be proteolytically cleaved into two parts, with the S1 subunit responsible for binding to angiotensin-converting enzyme 2 (ACE2) on the cell surface, and the S2 subunit responsible for the fusion of viral and cellular membranes (Fig. 2)^12^. The receptor binding domain (RBD), C-terminal domain of the S1 subunit, is immunodominant so that it is the major target of the neutralizing antibodies^13,14^. Morphological changes of S between pre- and post-fusion states have been characterized. In the pre-fusion state, the RBD exhibits its “up” conformation, allowing the RBD to bind to the receptor^15^. The S1 subunit is then released by furin cleavage at the boundary between S1 and S2^16,17^. Next, cellular transmembrane serine proteinase 2 (TMPRSS2) is recruited for viral priming by cleaving part of the S2 subunit upstream from the fusion peptide, resulting in the exposure of hydrophobic fusion peptide and insertion into host membranes^15,18,19^.

The vaccine is a powerful tool to tackle the pandemic. As of October 2022, WHO has listed twelve licensed vaccines for emergency use, the majority of which are produced based on the index virus (https://extranet.who.int/pqweb/vaccines/vaccinescovid-19-vaccine-eul-issued/). More than half of the countries have started programmatic implementation of booster vaccination, while some low-income countries are still trying to improve primary vaccination coverage (https://ourworldindata.org/coronavirus#explore-the-global-situation/). Despite these efforts, nearly three years since the outbreak of COVID-19, the causative SARS-CoV-2 is continuously evolving and generating variants. Existing vaccine-induced antibodies cannot fully neutralize the variants, especially the newly isolated Omicron variants^20–23^. For example, sera samples from two-dose BNT162b2 vaccine recipients show a 34-fold decrease of efficiency in inhibiting Omicron BA.1 spike entry compared with the D614G mutant strain^22^. Attention to emerging Omicron subvariants BA.4, BA.5, BF.7, BQ.1.1, and XBB is on the rise since they display distinct antibody evasion potentials^24,25^. There is an urgent need to develop universal vaccines with enhanced magnitude and breadth of neutralizing antibodies against emerging variants that show distinct immune escape abilities and even against other *Betacoronaviruses*. In this review, we summarize the reasons why universal vaccines for SARS-CoV-2 variants are difficult to produce and elaborate on several novel suggestions for SARS-CoV-2 universal vaccine designs.

## Challenges in universal vaccine design against SARS-CoV-2

The development of a new vaccine is a long-distance race between the virus and humans. Although tremendous progress has been made in reducing the viral transmission and fatality rate via vaccine administration, the emerging variants are challenging the protective efficacy of vaccines against symptomatic illness.

### High mutation rate of SARS-CoV-2 genome continually produces variants of concern

SARS-CoV-2 is a positive, single-stranded RNA virus that possesses the largest genome among the known RNA viruses that infect humans^26,27^. One notable feature of RNA viruses is their high mutation rate. Unlike DNA viruses which utilize the host replication machinery to detect and repair base-pairing errors during replication, RNA viruses use RNA-dependent RNA polymerases that lack proofreading ability and, therefore, are intrinsically error prone^28^. Although virus-encoded nonstructural protein 14 (nsp14) demonstrating exonuclease proofreading activity lowers SARS-CoV-2 mutation rate compared with other RNA viruses, SARS-CoV-2 variants accumulate over time^29^. SARS-CoV-2 is estimated to have 0.8–2.38 × 10^−3^ nucleotide substitutions per site per year, which is within the range of RNA virus mutation rate, but is one or more orders of magnitudes higher than that of DNA viruses^30,31^. High sequence variability of the S protein is a major obstacle in developing vaccines conferring broad protection.

**Fig. 1:**
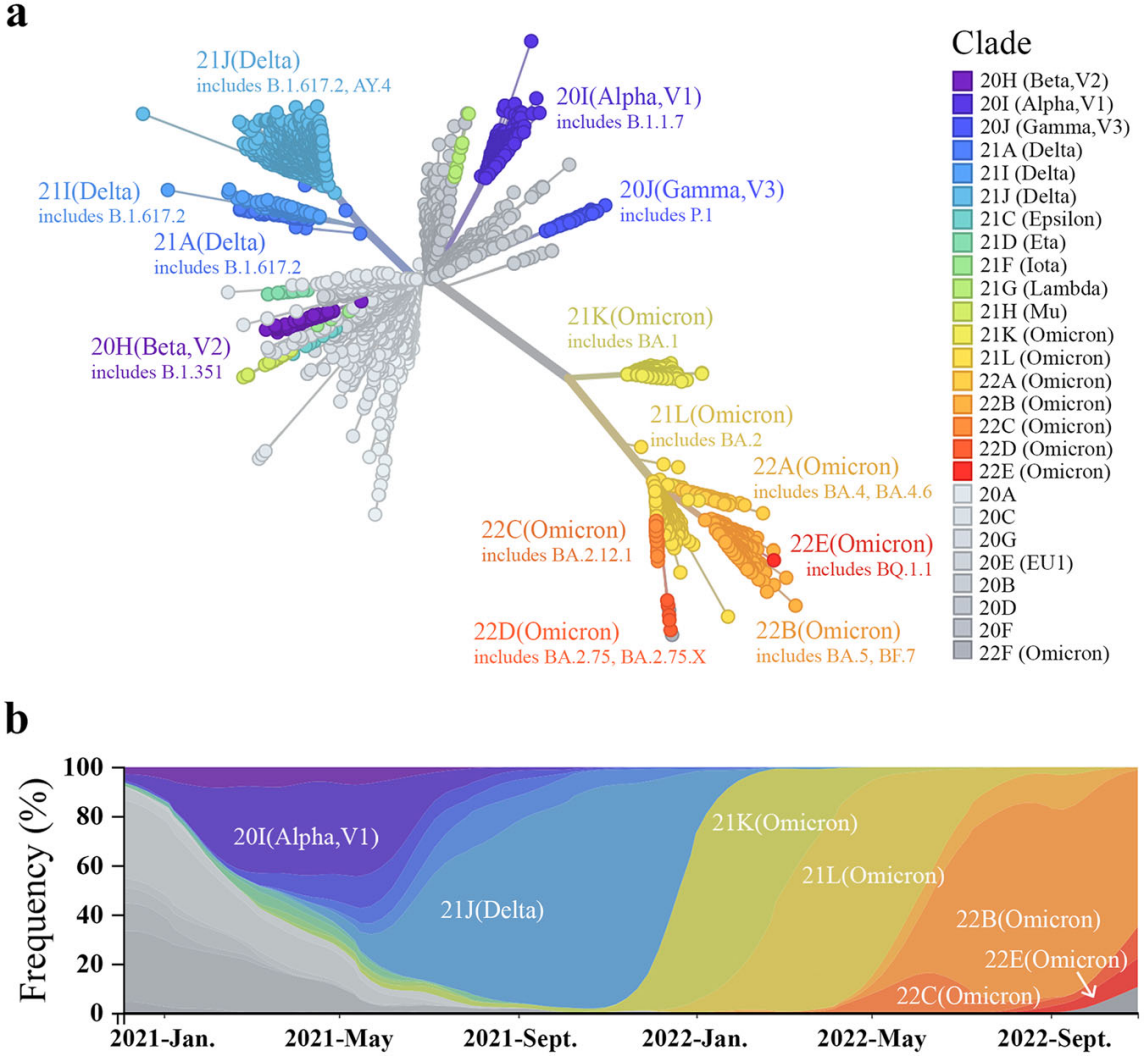
Circulating strains of SARS-CoV-2 are fast-changing. **a** Genetic relationships of global SARS-CoV-2 strains. The unrooted phylogenic tree is constructed by Nextstrain according to the sequences of 3050 SARS-CoV-2 spike glycoproteins from GISAID collected between December 2019 and October 2022 (https://nextstrain.org/ncov/gisaid/global/6m?l=unrooted&m=div). The tree is colored by clade and branch length represents the divergence of each clade. The Omicron variants show the highest antigenic distinction to the ancestral strain. **b** Time course of prevalent SARS-CoV-2 variants substitutions worldwide. The *x*-axis represents the date and the *y*-axis shows the frequencies of viral strains in percentage. Omicron subvariants have rapidly outcompeted previously prevalent Delta variants and become dominant.

SARS-CoV-2 variants with higher transmissibility, or other distinct characteristics that seriously affect public health are designated Variants of Concern (VOCs), namely Alpha, Beta, Gamma, Delta, and Omicron (https://www.who.int/en/activities/tracking-SARS-CoV-2-variants/) (Fig. 1a). The Alpha variant, firstly detected in early Autumn 2020, was more transmissible and caused more severe cases than the index virus^32,33^. Both the Beta and Gamma variants can escape from naturally acquired and vaccine-induced immunity^34–36^. The Delta variant emerged in India at the end of 2020 and caused a surge in confirmed cases^37^. The S protein of the Delta variant evolved to optimize cell entry by accelerating membrane fusion^38^. The Omicron variant outcompeted the previously prevalent Delta variant and became dominant worldwide soon after its discovery due to its higher transmissibility (Fig. 1b)^39,40^. According to WHO epidemiological reports, the Omicron BA.5, BA.4, and BA.2.75 descendent lineages remain the dominant strains circulating globally (https://www.who.int/emergencies/diseases/novel-coronavirus-2019/situation-reports).

### Characteristics of SARS-CoV-2 variants leading to immune escape

**Fig. 2:**
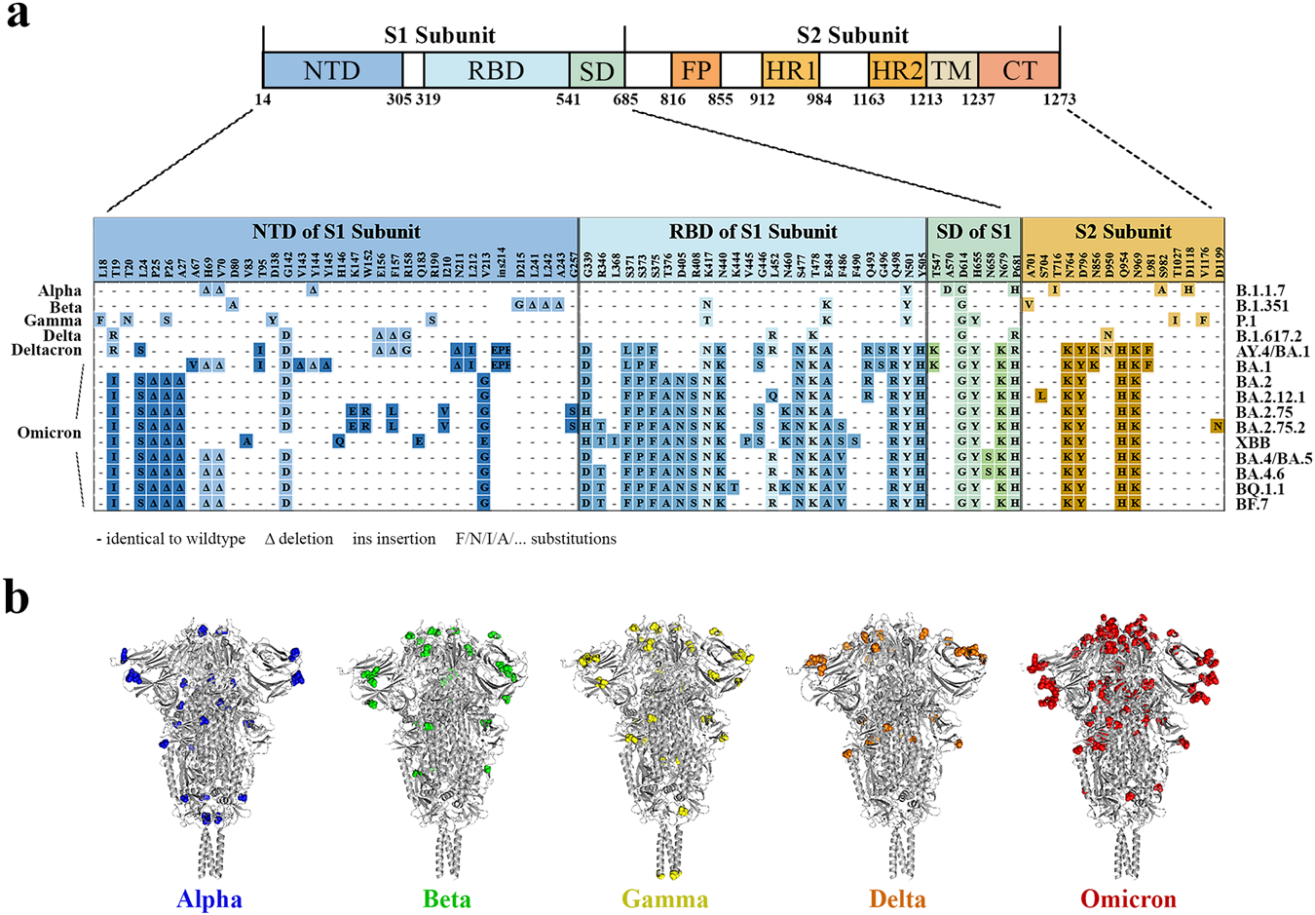
Composition of SARS-CoV-2 S and alignment of S mutation points of SARS-CoV-2 variants. **a** S can be proteolytically cleaved into S1 and S2 subunit. The S1 subunit contains the immunodominant N-terminal domain (NTD) and receptor-binding domain (RBD) as well as other subdomains. Fusion peptide (FP), heptad repeat 1 (HR1), heptad repeat 2 (HR2), transmembrane domain (TM), C-terminal peptide (CP) together with other unnamed parts comprise the S2 subunit. Names of the variants are labeled on both sides of the table. Mutation points are distinguished by different colors. Changes in Omicron-related strains are labeled with darker colors. Amino acid sequence of the index virus is included as a reference. At each given point, a hyphen means the amino acid is identical to that of the reference, Δ stands for deletion, ins stands for insertion, and various capital letters indicate substitutions. **b** Spatial positions of mutations in Alpha, Beta, Gamma, Delta, and Omicron variants are highlighted in structure models (PDB 6XR8).

Subtle structural differences of S protein in variants may facilitate viral immune escape, making it difficult to develop S-based universal vaccines^41,42^. The alignment of mutation sites of prevalent variants is shown in Fig. 2. The Beta and Omicron variants hold significant signs of immune escape, so the structural dynamics of these two variants will be introduced in detail.

There are only ten amino acid changes in the S protein of the Beta variant (Fig. 2), but it is reported to have an increased risk of immune escape according to testing showing nondetectable neutralizing activities in three classes of antibodies and convalescent plasma, as well as a 10.3- to 12.4-fold decrease in antibody neutralizing activities elicited by mRNA vaccines^43,44^. The L18F and D80A mutations produce a structural rearrangement of the N-terminal segment, while the triple deletion (L242del, A243del, and L244del) shifts segment 172-188 up 17 angstroms, affecting the interaction of antibodies that bind to this loop region^45^. A few hotspots during mutation accumulation can also be found in the Beta variant. Lys417 is crucial for interacting with a negatively charged residue in neutralizing antibodies; thus, a K417N mutation disturbs a salt bridge that forms during binding and leads to immune escape^46^. The E484K mutation results in both steric clashes and a charge change in the antibody-binding site in the receptor binding motif (RBM) region, which is considered to be an effective modification that reduces neutralizing sensitivity^44,47^.

The Omicron variant carries more than thirty mutations in the S protein and half of these changes are located in the antigenically important RBD region (Fig. 2)^48,49^. Even the sera from those who have recovered from COVID-19 or are vaccinated and boosted have poor neutralizing activities against the Omicron variant^50–53^. Critically, the Omicron variant causes less severe illness and fewer intensive care unit admissions compared with the Delta variant, indicating a long-term coexistence with infected or asymptomatic infected persons^54^. Several representative changes that can alter the antigenic characteristics of Omicron were found. For example, mutation G142D together with deletion 143–145 causes the N3 loop to change from a hair-pin shape to a loose loop, which in turn affects the conformation of the N4 and N5 loop^55^. This finding explains why Omicron has resistance to NTD-targeting neutralizing antibodies. Disruption of hydrophobic environment by G446S and induction of steric clash by N440K also reduce antibody binding affinities dramatically^46,55^. In addition, a previously unidentified combination of immune escape sites was described; mutations in S371L, S373P, and S375F cause a dramatic conformational change in the loop region that connects α2 and β2^55^. Remarkably, this antigenic site is always conserved in other *Sarbecoviruses*. Unique mutations exist in newly-isolated Omicron subvariants are dodging the immune system. BA.2.75 carries nine additional mutations in spike compared with BA.2, among which the D339H substitution causes a neutralization reduction^56^. One noteworthy mutation of BA.4/BA.5 in S is F486V, of which the location is near to S-ACE2 interaction site which is the main target for neutralizing antibodies^57^. In Omicron sublineages BA.1, BA.2, and BA.2.12.1, mutation Q493R with a longer side chain induces steric clashes that directly resist binding of antibodies, while reversion mutation R493Q in BA.2.75 and BA.4/BA.5 subvariants show opposite consequence, which may be a compromise strategy to confer more significant immune evasion together with other mutations than Q493R could have alone^51,55,57,58^. Another important mutation point R346T found in three Omicron subvariants BA.2.75, BA.4.6, and BF.7 (also known as BA.5.2.1.7) had shown extensive antibody-binding evasion and they appear to have increased prevalence over current dominant BA.4 and BA.5^59–62^.

The findings above suggest that high mutation rates provide SARS-CoV-2 with broader chances to survive via immune evasion, and the intension of mutation post great challenges to current universal vaccine studies and therapeutic antibody productions.

## Novel approaches in universal vaccine design against SARS-CoV-2

To date, vaccination is still the most effective method for reducing the morbidity and mortality rate, especially in the immunocompromised population that may facilitate viral evolution. The composition of the currently available COVID-19 vaccines needed to be updated in compensate of lower protection against symptomatic diseases and Omicron infection. The design of variant-specific vaccine candidates is more complicated. Apart from evaluating the immunogenicity and safety of the variant-specific vaccine candidate itself, more data is needed on efficacy, durability, and interval time in combination with currently available vaccines as a booster dose. Here, we list several important achievements, from clinically approved vaccines to vaccine designs that have shown broad-neutralizing abilities. We hope these findings will bring inspiration to current universal vaccine studies.

### Heterologous prime-boost regimens

The neutralization activity of antibodies induced by vaccination wanes gradually over time^63,64^. Furthermore, the emergence of new variants with stronger immune escape abilities increases the need for those who have received a COVID-19 primary series to get booster shots that contain updated vaccine compositions. Actions such as optimizing the use of currently available vaccines and increasing the protective efficacy of vaccines against evolving variants with booster vaccines are highlighted by WHO. Compared with homologous vaccination, mixing vaccines from different platforms or different SARS-CoV-2 strains may evoke greater cellular and humoral immune responses and achieve broader immunity against emerging variants.

Several heterologous booster administrations using licensed vaccines or vaccines in clinical trials have been widely implemented (Table 1). Heterologous SARS-CoV-2 vaccine strategies can be grouped by vaccine platforms. One popular combination at this stage is an adenovirus-based vector vaccine followed by boosting with an mRNA vaccine, which has been proven to induce strong cellular and humoral immune responses^65–70^. Neutralizing antibodies against the Alpha, Beta, and Gamma variants were induced by vaccinating participants with single dose of replication-deficient chimpanzee adenoviral vector (ChAdOx1) nCoV-19 vaccine and boosting with a mRNA vaccine BNT162b2^71^. In reverse, heterologous boosting with Ad26.COV2.S after BNT162b2 prime vaccination can also induce durable humoral and cellular immune responses^72^. An inactivated vaccine plus a recombinant adenovirus vectored vaccine or a recombinant protein subunit vaccine or a mRNA vaccine are additional heterologous vaccine regimens^73–77^. For example, initial vaccination with two doses of CoronaVac followed by heterologous boosting with the Ad5-nCoV induced significantly higher neutralizing antibody levels against the index virus and the Delta variant than did homologous boosting (5.9-fold and 6.7-fold, respectively)^73^. Cross-neutralizing antibody titers to the Delta or Omicron BA.4/5 variant elicited by aerosolized Ad5-nCoV booster vaccine is 18.1–24 or 7.88 times higher than that elicited by homologous booster vaccine^74,78^. Moreover, inhalation of aerosolized Ad5-nCoV elicited IgA and resident memory B and T cells around the mucosal area, which provided an effective antiviral barrier at respiratory tracks^74^.

**Table 1:** Combination of currently available heterologous vaccines.

Vaccine type	Vaccines (prime plus boost)	Manufacturer	Efficacy against variants (compared with homologous boosting)	References
Adenovirus vectored vaccine plus mRNA vaccine	ChAdOx1 nCoV-19 plus BNT162b2	AstraZeneca-Oxford/ Pfizer-BioNTech	Significantly higher nAb titers against Alpha, Beta and Gamma variants 17 days after booster shot	^71^
	Ad26.COV2.S plus BNT162b2 or mRNA-1273	Johnson & Johnson-Janssen/ Pfizer-BioNTech/ Moderna	2 times higher efficacy against symptomatic infection by Omicron 14 days to 1 month since last dose	^70^
	BNT162b2 plus Ad26.COV2.S	Pfizer-BioNTech/ Johnson & Johnson-Janssen	More durable humoral and cellular responses at least 16 weeks after boosting vaccination	^72^
Inactivated vaccine plus adenovirus vectored vaccine	CoronaVac plus Ad5-nCoV	Sinovac/ CanSino	6.8-fold higher levels of nAb titers against Delta variant 14 days after boosting	NCT04892459^73^
	CoronaVac plus aerosolised Ad5-nCoV		18.1–24 or 7.88 times higher nAb titers against Delta or Omicron variant as well as elicited IgA and resident memory B and T cells 28 days after booster dose	NCT05043259^74,78^
Inactivated vaccine plus recombinant protein subunit vaccine	CoronaVac or BBIBP-CorV plus ZF2001	Sinovac/ Sinopharm/ Zhifei Longcom	Induced at least 70-fold increase in neutralizing levels against Alpha, Beta, Gamma and Delta pseudoviruses 2 weeks after booster vaccination	^75^
Inactivated vaccine plus mRNA vaccine	CoronaVac plus BNT162b2	Sinovac/ Pfizer-BioNTech	6.3-fold increased nAb titers against Delta variant and 1.4-fold against Omicron 28 days after booster shot	^76^
	CoronaVac or BBIBP plus AWcorna	Sinovac/ Sinopharm/ Walvax	Higher neutralization antibodies against Delta and Omicron variants (6.5-times and 4.4-times, respectively) 28 days after booster	NCT04847102^77^

Another approach to combat the potential immune escape of SARS-CoV-2 variants is to design variant-specific booster vaccines (Table 2). Apart from one mRNA vaccine targeting both the index virus and the Omicron BA.1 variant from BioNTech was recently issued emergency use listing by WHO, bivalent COVID-19 mRNA vaccines (mRNA-1273.222 and Omicron BA.4/5 bivalent) from Moderna and Pfizer-BioNTech targeting SARS-CoV-2 Omicron subvariants BA.4 and BA.5 in addition to index virus have been authorized for emergency use as a booster dose by the U.S. Food and Drug Administration (FDA). Neutralizing BA.4/5 titers increase by more than 4- and 2.6-fold respectively after booster dose compared with their parental, monovalent vaccines mRNA-1273 and BNT162b2^79^. Different neutralizing performance may due to primary doses and the interval time between booster injection and bleeding. Immuno-bridging trial of these bivalent vaccines is recommended to further access their protective efficacy against the same subvariant. Heterologous boosting with Beta adjuvanted vaccine MVB.1.351 from Sanofi-GSK resulted in at least 1.5-fold higher antibody responses against index SARS-CoV-2, Beta, Delta, and Omicron BA.1 variants than boosting with a homologous BNT162b2 vaccine or a recombinant protein vaccine based on the original strain (NCT05124171)^80^. Omicron-specific inactivated vaccine candidates from China National Pharmaceutical Group Co. Ltd. (Sinopharm) and Sinovac Biotech Ltd. were also approved for clinical studies (NCT05374954 and NCT05381350) among adults who have received two or three doses of COVID-19 wild-type inactivated vaccines.

**Table 2:** Variant-targeting booster vaccines.

Vaccine type	Vaccines	Manufacturer	Phase & study description	Administration	Reference
SARS-CoV-2 S based mRNA vaccine	mRNA-1273 (ancestral strain), mRNA-1273.213 (Beta and Delta), mRNA-1273.211 (ancestral strain and Beta), mRNA-1273.617.2 (Delta), mRNA-1273.529 (Omicron), mRNA-1273.214 (ancestral strain and Delta variant), mRNA-1273.222 (ancestral strain and Omicron BA.4/BA.5)	Moderna	Phase 2/3 or completed; a study to evaluate the immunogenicity and reactogenicity of mRNA-1273.214 as booster vaccine	Prime: mRNA-1273; Boost: mRNA-1273.213/ mRNA-1273.211/ mRNA-1273.617.2/ mRNA-1273.529/ mRNA-1273.214/ mRNA-1273.222	NCT04927065^79^
	BNT162b2 (ancestral strain), BNT162b5 Bivalent (ancestral strain/ Omicron BA.2), BNT162b2 Bivalent (ancestral strain/ Omicron BA.1), BNT162b2 Bivalent (ancestral strain/ Omicron BA.4/BA.5)	Pfizer-BioNTech	Phase 2/3 or completed; an interventional randomized, active-controlled study to investigate the safety and immunogenicity of bivalent BNT162b RNA-based vaccine candidates as a booster dose	Prime: BNT162b2; Boost: BNT162b5 Bivalent/ BNT162b2 Bivalent (WT/OMI BA.1)/ BNT162b2 Bivalent (WT/OMI BA.4/BA.5)	NCT05472038
Recombinant S protein nanoparticle vaccine	NVX-CoV2373 (prototype-targeted) & NVX-CoV2515 (Omicron-targeted)	Novavax	Phase 3; a randomized, observer-blinded study to evaluate the safety and immunogenicity of 2 booster doses of the monovalent and bivalent vaccines adjuvanted with Matrix-M^TM^ adjuvant	Prime: other COVID-19 vaccines; Boost: NVX-CoV2515 or NVX-CoV2373 & NVX-CoV2515	NCT05372588
Protein subunit vaccine	MVD614 (monovalent formulation based on the original strain), MVB.1.351 (monovalent Beta formulation)	Sanofi-GSK	Phase 3; a study to evaluate immunogenicity and reactogenicity of two adjuvanted vaccines administered as booster dose in adults who received 2 doses of BNT162b2 before	Prime: BNT162b2; Boost: BNT162b2/ MVD614/ MVB.1.351	NCT05124171^80^
	SCTV01C (Alpha and Beta variants S-trimer vaccine), SCTV01E (Alpha/Beta/Delta/Omicron variants S-trimer vaccine)	Sinocelltech	Phase 3; a randomized, double-blind, positive-controlled study to evaluate the immunogenicity and safety of one dose SCTV01C or SCTV01E as a booster compared with homologous booster in adults	Prime: BBIBP/ mRNA-1273; Boost: BBIBP/ mRNA-1273/ SCTV01C/ SCTV01E	NCT05308576, NCT05323461^81^
Protein subunit vaccine	V-01-351 (Beta-targeted) & V-01D (Delta-targeted)	Livzon	Phase 2; a single arm, open label study to evaluate the immunogenicity and safety of bivalent vaccine in healthy adults who have vaccinated with 2 doses of inactivated vaccines	Prime: inactivated COVID-19 vaccine; Boost: V-01-351 & V-01D	NCT05273528^82^
Omicron-based inactivated vaccine	Omicron COVID-19 Vaccine	Sinopharm	Phase 3; a randomized, double blind, positive controlled study in population aged 18 y and above who have received 2 or 3 doses of inactivated COVID-19 vaccines	Prime: prototype inactivated vaccine; Boost: Omicron variant inactivate vaccine	NCT05374954
		Sinovac	The same as above	Prime: inactivated vaccine (CZ strain); Boost: inactivated vaccine (Omicron variant)	NCT05381350

Other variant-updated candidate vaccines have entered different phases of clinical trials. A phase 3 clinical study has been activated to test the safety and immunogenicity of a two booster doses of the recombinant protein nanoparticle vaccine (NVX-CoV2373 for prototype plus NVX-CoV2515 for the Omicron variant) from Novavax (NCT05372588). SCTV01C, which is designed based on the extracellular domain of trimeric S of the Alpha and Beta variants, is found to have cross-neutralizing activities against not only the Alpha and Beta variants but twelve other variants^81^. An updated COVID-19 Alpha/Beta/Delta/Omicron variants S-trimer vaccine named SCTV01E from the Sinocelltech Ltd. elicits 1.4-fold higher neutralizing titers against Omicron BA.1 and BA.5 than SCTV01C in a phase 3 clinical trial study (NCT05308576, NCT05323461). Another protein subunit bivalent vaccine V-01-351/V-01D produced by Livzon Pharmaceutical Group Inc. comprises two variant-targeted antigens (V-01-351 for the Beta variant and V-01D for the Delta variant). This vaccine candidate showed higher neutralizing antibody titers against the Omicron variant as a booster dose compared with monovalent prototype or variant reference vaccines (NCT05273528)^82^.

### Chimeric immunogens

**Fig. 3:**
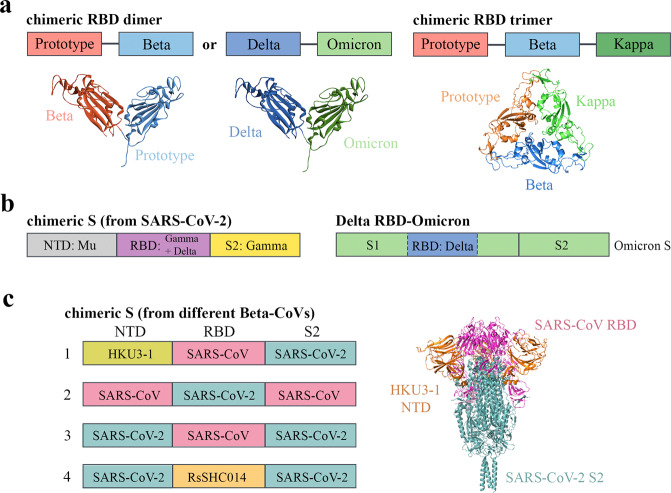
Schematic demonstration of chimeric antigen design. **a** Combinations of chimeric RBDs. Prototype-Beta chimeric RBD-dimer is a tandem repeat single chain dimer built with one prototype RBD and one Beta RBD. Similarly, Delta-Omicron RBD-dimer is built with one Delta and one Omicron RBD. For NVSI-06-08 or chimeric RBD trimer, three RBDs were truncated from prototype, Beta and Kappa variants and connected end-to-end. Structural arrangements of chimeric RBD dimers or trimer are shown in lower panel. Each monomer is marked by the same color as that shown in the upper panel. **b** Antigenic S ectodomain formed by building blocks from different SARS-CoV-2 strains. The recombinant monomeric spike variant (STFK1628x, annotated as chimeric S) contains the NTD from Mu variant, RBD-S2 from Gamma variant and RBD region is additionally patched by mutations from Delta variant. For the Delta RBD-Omicron chimera design, the Omicron spike is used as a backbone and the Delta RBD is inserted directly upstream of the Omicron RBD. **c** There are four spike chimera constructs from different Beta-CoVs in total. Spike chimera 1 contains an HKU3-1-derived NTD, SARS-CoV-derived RBD, and SARS-CoV-2-derived S2. Spike chimera 2 contains a SARS-CoV-2-derived RBD, and the NTD and S2 are of SARS-CoV origin. The origins of the building blocks in spike chimera 3 are opposite to that of spike chimera 2. Spike chimera 4 includes an RBD from RsSHC014 and the rest are from SARS-CoV-2. A model of chimera 1 is generated by PyMOL based on a previously solved cryo-EM structure (PDB 6XR8).

Protein subunit vaccines are widely promoted because of their simple formulation. Monomeric, dimeric, and trimeric RBD subunit vaccines appear to be immunogenic and well-tolerated, but their efficacy to SARS-CoV-2 variants is found dissatisfactory^83–86^. Another novel method by which antigens elicit cross-protective antibodies assembles key immunogens from different SARS-CoV-2 strains into a single protein subunit. Chimeric antigen vaccine candidates that contain either prototype-Beta or Delta-Omicron RBD dimer were designed (Fig. 3a)^87^. Both chimeric protein subunit vaccines elicited greater breadth in the neutralizing activities against SARS-CoV-2 variants than homotypic prototype dimer or variant-specific dimer^87^. A vaccine named NVSI-06-08 (annotated as mul-tri-RBD in Fig. 3a) containing a mutation-integrated trimeric form of RBDs was generated^88^. The hybrid immunogen formed by prototype, Beta, and Kappa RBDs induced significantly higher titers of neutralizing antibody responses against the Beta and Delta variants than a homotrimer constructed from the prototype strain alone^88^.

The idea of chimeric immunogen construction can also be applied to the complete spike ectodomain. A bivalent vaccine candidate (prototypic STFK plus STFK1628x) can elicit high titers of broad-spectrum antibodies against the majority of the circulating SARS-CoV-2 variants^89^. STFK1628x (annotated as chimeric S in Fig. 3b) is a monomeric spike ectodomain protein that harbors several mutations gathered from different variants using inter-lineage chimera and mutation patch strategies: B.1.620-derived NTD and Gamma-derived RBD-S2, with RBD additionally patched by mutation points from the Delta variant^89^. Recently, a chimeric immunogen called Delta RBD-Omicron was constructed by inserting the Delta variant RBD upstream of Omicron RBD in an Omicron spike skeleton (with furin cleavage site mutation) (Fig. 3b)^90^. Compared to mice immunized with Omicron Spike mRNA, the neutralizing antibody titers elicited by this immunogen were significantly higher against the Delta variant and similar to the Omicron variants, including BA.1, BA.2, and R346K mutation^90^.

These designs provide a complementary approach to broaden the cross-variants antigenic coverage. Ideas such as these can be applied to develop universal vaccines against the *Sarbecovirus* subgenus. For example, the mRNA sequences of chimeric spikes that encode the NTD, RBD, and S2 domains from SARS-CoV, SARS-CoV-2, and bat CoV were gathered in different combinations (Fig. 3c)^91^. These mRNA-lipid nanoparticle vaccines containing chimeric spikes induced high levels of broad-neutralizing antibodies (BnAbs) against several *Sarbecovirus* strains.

### Protein nanoparticle antigens

Chimeric antigens do provide cross-reactive protection against SARS-CoV-2 variants. However, some antigens are too small to raise robust immune responses, novel techniques need be applied to ensure that protein subunits are capable of being captured and presented by the host immune system. Nanoparticles with protein antigens embedded on the surface facilitate drainage to lymph nodes and enhance the possibility they will be taken up by antigen-presenting cells, leading to a more robust neutralization response and higher cross-reactive antibody protection compared with the antigen alone^92–95^. Self-assembling nanoparticles together with immunogenic epitopes (RBD or full-length S protein or their modified versions) elicited long-lasting antibody responses against SARS-CoV-2 and its VOCs, and with persistent memory B cells^96–99^. NVX-CoV2373 is a recombinant nanoparticle vaccine that is composed of trimeric S and Matrix-M^TM^ adjuvant. Two doses of NVX-CoV2373 can elicit immune responses against SARS-CoV-2 Alpha and Beta variants^100–102^. Joyce et al. developed a spike ferritin nanoparticle (SpFN) vaccine for SARS-CoV-2 using the S sequence of index virus. The adjuvanted vaccine was confirmed to induce a Th1-biased cellular response and elicited neutralizing antibodies against original SARS-CoV-2, Alpha, Beta, Gamma and Delta variants in nonhuman primates^99^. It has completed its phase 1 clinical trial at the end of December, 2021 (NCT04784767).

**Fig. 4:**
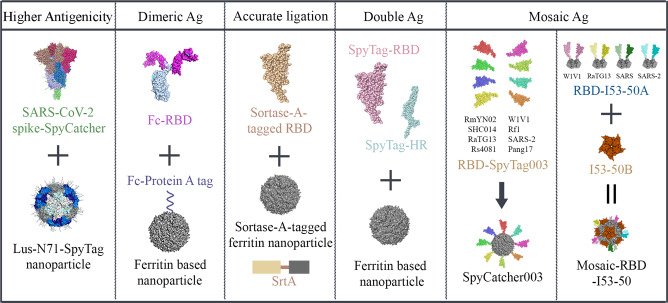
Overview of antigens presented by nanoparticles. Plug-and-play platform increases the efficiency of protein subunit vaccine production. *Aquifex aeolicus* lumazine synthase (LuS) is chosen as a scaffold to induce significantly higher neutralizing responses. The RBDs fused with an Fc tag forms an antigen dimer automatically, enabling rapid generation of vaccines targeting to variants. Sortase-A (Srt-A) is chosen for accurately ligating antigens onto nanoparticles. More than one antigen can be assembled onto the same ferritin nanoparticle using SpyTag, SpyTag003 or I53-50A, which can induce cross-reactive immunity against other coronaviruses or *Sarbecoviruses*. SARS SARS-CoV, SARS-2 SARS-CoV-2.

There are many technological breakthroughs in antigen-nanoparticle construction (Fig. 4). The plug-and-play platform, in which protein antigens and nanoparticle scaffolds can be produced simultaneously but independently, has been widely used as a versatile product to improve the inefficiencies in protein subunit vaccine production. *Aquifex aeolicus* lumazine synthase (LuS) is chosen as a nanoparticle scaffold in SpyCatcher/SpyTag antigen conjugation system to stimulate particularly potent neutralizing responses^92^. Antigen RBD can be presented in dimeric form automatically via fusing with a Fc tag^103,104^. The FNP-Fc-RBD_Omicron_ protects mice from infections by the BA.1 and BA.2 variants, offering great potentials to generate vaccines targeting other emerging variants^103^. For site-specific integration of antigen proteins to ferritin nanoparticles, Saunders et al. choose Sortase A reaction and found that this SARS-CoV-2 RBD-based nanoparticle vaccine candidate elicited high titers of cross-neutralizing antibodies against bat coronaviruses, SARS-CoV, and SARS-CoV-2 (including the Alpha, Beta and Gamma variants) in macaques^105^. In addition, SpyTag003/SpyCatcher003, an updated version of SpyTag/SpyCatcher system that overcomes the symmetry mismatch problem, is more thermostable and immunogenic and has been engineered for presenting RBDs on the surface through covalent isopeptide bonds^106,107^.

Efforts are not merely limited to generating larger immunogens. Research groups are evaluating various combinations that multivalently display the antigens on a single nanoparticle surface, which paves the way for developing pan-sarbecovirus and pan-betacoronavirus vaccines (Fig. 4)^108–113^. For example, mosaic-8 RBD nanoparticles made by Cohen et al. provide potential strategies to protect against emerging zoonotic coronaviruses and *Sarbecoviruses*^108,109^. The highly conserved heptad repeat (HR) region was incorporated into a nanoparticle vaccine candidate. RBD and HR fused with SpyTag were expressed and purified separately and were then covalently conjugated to *Helicobacter pylori* ferritin at a 7:3 ratio (ST-RBD to ST-HR), and the construct was found to reduce viral load in the lungs after SARS-CoV-2 challenge^110^. More importantly, neutralizing antibodies induced by RBD-HR nanoparticles contributed to cross-protective immunity against other coronaviruses^110^. Similarly, a bivalent nanoparticle vaccine candidate constructed by Yuan et al. via displaying the RBDs of the D614G and Beta variants onto ferritin nanoparticle can elicit cross-neutralizing antibodies against SARS-CoV-2 variants in rhesus macaques after three doses of infection^111^. Based on a computationally designed, symmetrical icosahedral I53-50 system, Walls et al. constructed a nanoparticle containing multivalent RBD epitopes^112^. Trimeric I53-50A and pentameric I53-50B constituted this system and RBDs were connected to multiple I53-50A using linkers comprising 8, 12 or 16 glycine and serine residues, ensuring a flexible spatial extension on the nanoparticle surface^112^. Pseudovirus and serum neutralization assay results showed that it elicited a 10-fold higher neutralizing antibody titer that recognized multiple RBD regions at very low injection doses compared to the one using a prefusion-stabilized S^112^. They further designed an I53-50A trimer-based RBD cocktail using RBDs from SARS-CoV, SARS-CoV-2 and two bat coronaviruses^113^. This multivalent RBD vaccine protected mice from a heterotypic *Sarbecovirus* challenge^113^.

### Conserved neutralizing epitopes

**Fig. 5:**
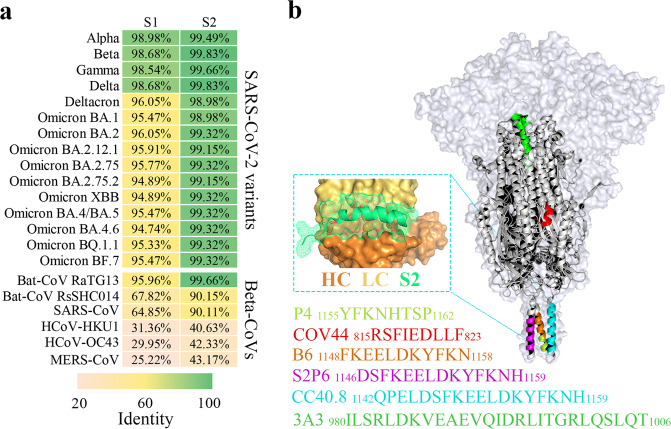
S2 is more conserved and has distinct neutralizing epitopes. **a** Heatmap diagram showing the S1 and S2 sequence identity of SARS-CoV-2 variants and other representative Beta-CoVs compared with the ancestral SARS-CoV-2 strain. Sequence information of each strain was obtained from the NCBI database and identity was generated automatically while blasting against the corresponding sequence from the reference. Pink, low identity; Green, high identity. **b** 3D structural model diagram presents the neutralizing epitopes of SARS-CoV-2 S2. The entire SARS-CoV-2 spike trimer is shown with the transparent molecular surface and S2 trimer is shown in a cartoon. Peptides that interact with isolated monoclonal antibodies are highlighted with different colors, with corresponding sequences and positions listed below. A zoomed-in view of the interaction between the CC40.8 antibody and the S2 stem-helix is shown in the cyan rectangle. The main chain of the S2 stem peptide is colored in green, while the heavy chain (HC) and light chain (LC) of the antibody are shown in orange and yellow, respectively. The S trimer model is generated by PyMOL based on a previously solved cryo-EM structure (PDB 6XR8). Zoomed-in view of the interaction between CC40.8 and stem helix is reconstructed by PyMOL (PDB 7SJS).

Progress in the isolation of broadly neutralizing antibodies among coronaviruses also provides an innovative approach in vaccine design. These antibodies target multiple conserved neutralizing epitopes in spike, especially S1-RBD and S2 regions. Although S1 in S is antigenically important and the S1-ACE2 interaction is crucial for viral entry, its high mutation rate contributes to viral escape from antibody neutralization^114–118^. S2, the stem region of the S homotrimer, is more conserved than S1 in terms of sequence and structure, which offers a novel path for universal COVID-19 vaccine design (Fig. 5a)^119^. Universal vaccine studies of influenza viruses have verified the effectiveness of antibodies elicited by the conserved stalk region of the surface glycoprotein hemagglutinin in inhibiting viral entry^120,121^. The latest study suggests that sera from SARS-CoV-2 S2 immunized mice can broadly neutralize different human and animal CoVs as well as ancestral SARS-CoV-2 strain and some VOCs^122^.

However, the coronavirus spike protein is covered by numerous N-linked glycans controlling protein correct folding^123^. Similar to SARS-CoV, the S2 subunit of SARS-CoV-2 is more decorated by N-linked glycan shielding than the S1^10^, which results in less accessibility for immune recognition and, therefore, low immunogenicity^124^. Besides, conformational changes around fusion peptide that is initiated by S1 cap removal are needed for mAb binding^125^. Thus, using conserved epitopes of the S2 subunit as potential targets in peptide-based universal vaccine development is a trade-off between breadth and efficacy.

Reported neutralizing antibodies targeting the S2 subunit of coronaviruses can be divided into two groups according to the hosts they were isolated from: mice origin, such as B6, B10 and 3A3, and patient origin, such as CC40.8, S2P6 and CV3-25^126–132^. These mAbs together cover distinct epitopes of the S2 subunit (Fig. 5b). The stalk region is the main target for S2-specific mAbs. In one recent study, Zhou et al. isolated a BnAb named CC40.8 from a SARS-CoV-2 convalescent^127^. CC40.8 exhibited effective neutralizing activities against SARS-CoV, SARS-CoV-2, and other *Betacoronaviruses*. Furthermore, CC40.8 also neutralize the SARS-CoV-2 Alpha, Beta, Gamma and Delta variants, which is a consequence of a highly conserved 25-residue peptide within the S2 stem helix^127^. CC40.8 around the stalk region of S2 produces a high atom density that may hinder protein refolding from the pre- to post-fusion state, and thus block viral entry. The binding motifs of B6, S2P6 and CV3-25 mAb overlap with the epitopes of CC40.8^126,128,129^. In addition, Dacon et al. identified several mAbs (named COV44) binding to the fusion peptide region of the S2 subunit which is located immediately adjacent to the S2’ cleavage site and is highly conserved among four coronavirus genera^125^. The 3A3 mAb binds the apex of the S2 domain which is distal to the viral envelope and the binding is influenced by the RBD position^131^.

Utilization of these conserved neutralizing epitopes discovered in S2 represents opportunities for universal vaccine development. The broadly neutralizing peptide P4, which is located at the stalk region of S2, is conserved among BatCoV-RaTG13, SARS-CoV, SARS-CoV-2, and its variants.^132^. Sera from mice immunized with the purified trimeric peptide P4 effectively neutralized SARS-CoV-2 S-pseudovirus, indicating its potential value as a universal vaccine candidate against SARS-CoV-2^132^. Some other broad-spectrum neutralizing epitopes, including S2’ cleavage site and the apex of the S2 domain, also deserve further exploration as broad-spectrum vaccine targets.

## Conclusions and future perspectives

Since the outbreak of the COVID-19 pandemic, a great many groups have conducted rapid vaccine studies. The experience accumulated from vaccine studies for coronavirus strains such as SARS-CoV and MERS-CoV support the development of SARS-CoV-2 vaccines. Unlike most vaccines that require more than ten years to launch, accelerated production and delivery of SARS-CoV-2, to some extent, help reduce the mortality rate. However, new variants with uncertain evolutionary directions are constantly reported, which is directly linked to unpredictable epidemiology of SARS-CoV-2 and thus hinders the progress of terminating the global pandemic. We suggest to expedite regulatory approval to develop and produce pan-SARS-CoV-2 or pan-betacoronavirus. Councils like the Coalition for Epidemic Preparedness Innovation (CEPI) have announced to provide more fundings on pan-betacoronavirus vaccine creation. More vaccine manufacture groups are turning their sights on emerging variants or vaccines designs that can provide broader protection.

**Fig. 6:**
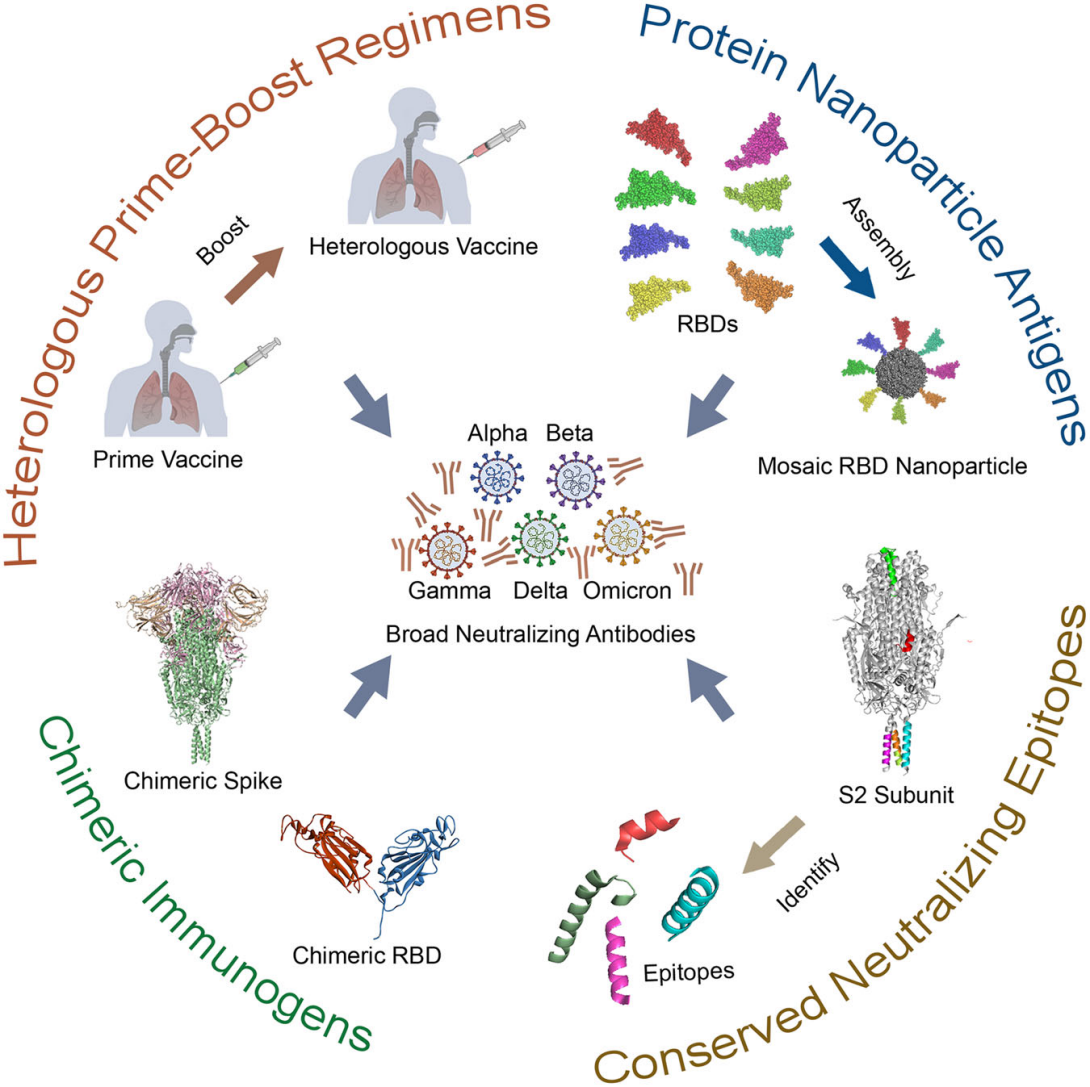
Four strategies in SARS-CoV-2 universal vaccine design. They are heterologous prime-boost vaccination regimens, construction of protein nanoparticle antigens, design of chimeric immunogens, and utilization of conserved neutralizing epitopes.

In this review, we speculate that the limited development of universal SARS-CoV-2 vaccines is highly related to frequent mutations that occur in the SARS-CoV-2 genome and the resulting structural reshaping of major epitopes. Four advances from distinct fields that widen neutralizing antibody protection against variants were summarized, including heterologous prime-boost vaccination regimens, construction of chimeric immunogens, design of protein nanoparticle antigens, and utilization of the conserved neutralizing epitopes (Fig. 6). Among all the approaches mentioned above, heterologous prime-boost regimen is a ready-to-use but passive or temporal hysteresis method in response to variants. That is to say, only when new variants occur, variant-specific vaccines will be produced. By comparison, chimeric antigen constructions and antigen presented by nanoparticles show superiority in combating emerging variants by assembling different immunogens or conserved neutralizing epitopes into a single particle. The main technique used in these two methods is purification of recombinant protein subunits, which enables large-scale production and, at the same time, emphasizes the importance of building a favorable protein expression system. Although less sensitive in inducing effective neutralizing antibodies as RBD or S does, the neutralizing epitopes discovered in S2 show great exploration value to serve as targets for universal vaccines, which has been proven to be conserved among coronaviruses or at least within SARS-CoV-2 variants. We highly expect new vaccines from the later three novel approaches could enter clinical trials for further investigation.

This review does not cover all the developments in universal vaccine design of SARS-CoV-2, other novel ideas and updated clinical trial results are reported continually. Universal vaccine design is not restricted to elicitation of neutralizing antibodies, new aspects that can provide broader immune responses, for example, vaccines generating robust cellular responses, are encouraged^133,134^. Protein subunit vaccines designed based on conserved T-cell epitopes from S, Nucleocapsid (N), and Membrane (M) proteins show high potential value as universal vaccines^135,136^. Novel adjuvants not listed here offer great help as well^100,137–139^. Furthermore, intramuscular administration of vaccines are less likely to block viral transmission at upper respiratory tracts^140^. Vaccinees can benefit from the induction of a mucosal immune response (mainly sIgA) in respiratory tracts by a nasal vaccine that mimics the natural infection process^141^.

SARS-CoV-2 vaccines have upgraded from the traditional inactivated to genetically engineered vaccines including protein subunit vaccines, DNA/mRNA vaccines and viral vectored vaccines. They have witnessed the advancements that each generation of vaccines has made, but they still have not accomplished the goal of defeating the COVID-19 pandemic. WHO and other entities have urged that it is critically important to proceed with the development of pan-SARS-CoV-2 vaccines or vaccines that might stimulate BnAbs against multiple coronaviruses in human bodies. Work in universal vaccine development should also be extended to other infectious diseases that may pose pandemic threats. If we prepare in advance, the production time for effective vaccines will be reduced and more lives will be saved.

## Data Availability

Data sharing is not applicable to this review since no data was generated or analyzed.
